# Evaluation of a widely used culture-based method for detection of livestock-associated meticillin-resistant *Staphylococcus aureus* (MRSA), Denmark and Norway, 2014 to 2016

**DOI:** 10.2807/1560-7917.ES.2017.22.28.30573

**Published:** 2017-07-13

**Authors:** Jesper Larsen, Marianne Sunde, Md Zohorul Islam, Anne Margrete Urdahl, Aina Steihaug Barstad, Anders Rhod Larsen, Carl Andreas Grøntvedt, Øystein Angen

**Affiliations:** 1 Statens Serum Institut, Copenhagen S, Denmark; 2 These authors contributed equally as first authors; 3 The Norwegian Veterinary Institute, Oslo, Norway

**Keywords:** meticillin-resistant Staphylococcus aureus, MRSA, livestock, laboratory surveillance, detection, sensitivity

## Abstract

We evaluated a widely used culture-based method for detection of livestock-associated meticillin-resistant *Staphylococcus aureus* (LA-MRSA) in samples collected from pigs and the environment inside pig stables in Denmark and Norway. Selective enrichment in tryptic soy broth containing cefoxitin and aztreonam led to a high ratio of false-negative results (26%; 57/221). On this basis, we recommend reconsidering the use of selective enrichment for detection of LA-MRSA in animal and environmental samples.

The recommended method for detection of livestock-associated meticillin-resistant *Staphylococcus aureus* (LA-MRSA) includes two enrichment steps (hence referred to as the 2-S method). The second enrichment broth contains 3.5 or 4 mg/L cefoxitin and 75 mg/L aztreonam. Since 2008, cefoxitin and aztreonam at these concentrations have been suspected to produce false-negative result [1]. Therefore, we evaluated the sensitivity of the 2-S method using samples collected from Danish and Norwegian pigs between 2014 and 2016, with special emphasis on the second enrichment step. This was done by comparing the 2-S method with an alternative method, where the selective enrichment step is bypassed (hence referred to as the 1-S method).

## Sample collection

Samples used to evaluate the two methods were collected from pigs and the environment inside pig stables in Denmark and Norway, countries with high and low levels of LA-MRSA in their pig populations, respectively. In Denmark, 136 pools of five cotton swabs taken from the anterior nares of individual pigs; these samples were a subset of samples collected by the Danish Veterinary and Food Administration, as part of a 2016 survey on LA-MRSA on pig farms [2]. In Norway, 1,958 pig and environmental samples were collected for active surveillance purposes and during outbreak tracing by the Norwegian Food Safety Authority over a 2-year period from December 2014 to January 2016 [3,4]. In brief, sterile cloths (Sodibox) were used to swab the skin behind the ears of animals (up to 20 animals per cloth) as well as indoor surfaces in the pig stables (up to 15 contact points per cloth).

## Comparison of the 2-S and 1-S methods

Pools of five cotton swabs, pools of two to three cloth swabs taken as part of the active surveillance programme, and individual cloth swabs collected during outbreak tracing were inoculated in 10 mL, 500 mL and 300 mL of Mueller-Hinton broth (MHB) containing 6.5% NaCl, respectively, and incubated for 16–24 h at 35–37 °C. A 10-µL loopful of pre-enrichment culture was spread on Brilliance MRSA 2 agar plates and incubated for 16–24 hour at 35–37 °C (1-S method). In addition, 1 mL of pre-enrichment culture was added to 9 mL of tryptic soy broth (TSB) containing 3.5 mg/L cefoxitin and 75 mg/L aztreonam, followed by incubation for another 16–24 h at 35–37 °C. Finally, a 10-µL loopful of selective enrichment culture was spread on Brilliance MRSA 2 agar plates and incubated for 16–24 hour at 35–37 °C (2-S method). Presumptive MRSA colonies were confirmed by PCR [5].

The 2-S method and the alternative 1-S method detected MRSA in 74% (100/136) and 82% (112/136) of the Danish samples and in 3.8% (74/1,958) and 5.6% (109/1,958) of the Norwegian samples, respectively (Table).

**Table:** Test results of the 2-S and 1-S methods for detection of livestock-associated meticillin-resistant *Staphylococcus aureus* in samples collected from pigs and the environment of pig stables, Denmark and Norway, 2014–2016 (n = 2,094)

Country	1-S	2-S		Total
		Positive	Negative	
Denmark	Positive	98	14	112
	Negative	2	22	24
	**Total**	**100**	**36**	**136**
Norway	Positive	66	43	109
	Negative	8	1,841	1.849
	**Total**	**74**	**1,884**	**1.958**
Combined	Positive	164	57	221
	Negative	10	1,863	1,873
	**Total**	**174**	**1,920**	**2,094**

The two methods yielded significantly different results, both in Denmark and Norway, and when the results from the two countries were combined (p = 0.0060 vs p = 0.0001 vs p = 0.0001 by 2-sided McNemar test).

The two methods generated identical results in 88% (120/136) and 97% (1,907/1,958) of the samples from Denmark and Norway, respectively. The remaining samples from Denmark (n = 16) and Norway (n = 51) produced different results with the two methods. The 2-S method failed to detect MRSA in 13% (14/112) of the Danish and 39% (43/109) of the Norwegian samples that yielded a positive result with the 1-S method. In contrast, the 1-S method only failed to detect MRSA in 2% (2/100) of the Danish and 11% (8/74) of the Norwegian samples that were positive for MRSA when using the 2-S method. 

Unfortunately, there is currently no appropriate gold standard for identification of LA-MRSA in biological specimens, which makes it difficult to assess and compare performance characteristics of the two methods (e.g. sensitivity, specificity, positive predictive value (PPV) and negative predictive value (NPV)). Instead, we compared the test results using the 2-sided McNemar test [6]. The analysis supported that the two methods yielded significantly different results, both in Denmark (p = 0.0060) and Norway (p = 0.0001). 

## Discussion

Livestock-associated meticillin-resistant *Staphylococcus aureus* (LA-MRSA) is an important cause of human disease in countries with a low overall level of MRSA in humans, such as Denmark and the Netherlands [7]. A 2008 survey by the European Food Safety Authority (EFSA) showed that LA-MRSA was well established in the pig populations of many European countries [8]. 

In Denmark, the prevalence of MRSA-positive pig farms has increased dramatically over the years, from 3.5% in 2008 to 88% in 2016 [2,8]. This uncontrolled epidemic spread has been accompanied by a rapid increase in the number of human infections, from 16 cases in 2008 to 207 cases in 2015 [9,10]. Historically, LA-MRSA has primarily been described as a cause of skin and soft tissue infections in farm workers, but it has also been associated with sepsis and even death in immunocompromised patients [11]. In contrast to Denmark, Norway has been able to maintain low levels of LA-MRSA in its pig population. In the latest survey from 2016, LA-MRSA was found in only one of 872 participating pig farms [12]. The reasons for this success can be explained by restricted import of live pigs and a control strategy which includes recommendations for targeted screening of personnel before working in pig herds, active surveillance of the pig population, and a ‘search and destroy’ policy on pig farms [13].

The European Union Reference Laboratory for Antimicrobial Resistance (EURL-AR) within the context of animal health and food safety recommends using the 2-S method for detection of LA-MRSA in dust swabs taken from the environment inside pig stables [14]. The 2-S method was first used in the 2008 survey [8] and includes two enrichment steps followed by plating on Brilliance MRSA 2 agar medium (Oxoid) and confirmation of presumptive MRSA colonies using PCR [5,14,15]. This method is also recommended by EFSA for detection of LA-MRSA in nasal swabs taken from pigs at the slaughterhouse or farm and in boot swabs taken at the farm [16]. The pre-enrichment step is performed in MHB containing 6.5% NaCl, whereas the selective enrichment step is performed in TSB containing 3.5 or 4 mg/L cefoxitin and 75 mg/L aztreonam. However, it has previously been shown that cefoxitin and aztreonam at concentrations of 4 mg/L and > 20 mg/L, respectively, can lead to false-negative result [1], which raises questions about the reliability of the EURL-AR method.

Our findings confirm that the 1-S method is more sensitive than the 2-S method (i.e. the 1-S method has a higher NPV and thus a lower ratio of false-negative results). In addition, it is cheaper and has a 24 h shorter turnaround time. Thus, findings based on the 2-S method should be interpreted with caution. For example, the prevalence of LA-MRSA at pig farms may have been underestimated in countries such as Denmark, where the 2-S method is used by the national reference laboratories for routine surveillance. It should be noted, though, that EURL-AR and EFSA recommend testing five samples from each pig farm [15,16], which in theory would at least partly compensate for the lower sensitivity of the 2-S method.

With both methods, samples collected in Norway were associated with a high ratio of false-negative results, compared with samples from Denmark. There are several possible explanations for this, including variations in the level of MRSA at the sampled pig farms and the use of different sampling techniques. In addition, there may be differences between the clonal structure of the Danish and Norwegian LA-MRSA populations and their tolerance to cefoxitin and aztreonam at the concentrations used in the 2-S method. Although we did not determine antimicrobial susceptibility or minimum inhibitory concentrations in our study, we do not believe this to be the case, as the vast majority of contemporary LA-MRSA isolates circulating in the Danish and Norwegian pig populations belong to the same clonal complex, CC398 [13,17]. Whole-genome sequencing analysis even showed that LA-MRSA CC398 isolates from the two countries are very closely related [13]. Finally, the presence of other bacteria in the samples may be different in the two countries, leading to different degrees of undesired overgrowth and outcompetition of LA-MRSA in the media used. This hypothesis is supported by unpublished findings from Norway, where the 1-S method sometimes leads to false-positive results due to growth of meticillin-susceptible *S*. *aureus* (data not shown), emphasising the importance of PCR confirmation of presumptive MRSA colonies.

## Conclusion

The 2-S method recommended by EURL-AR and EFSA for detection of LA-MRSA in samples collected from pigs and the environment inside pig stables led to a considerably higher ratio of false-negative results than the 1-S method. The performance of the two methods is likely to be influenced by the concentration and population structure of LA-MRSA and other bacteria in a given country, host species (pig, cattle, poultry, etc.), and environment. As a consequence, the results presented here cannot be directly extrapolated to analyses of samples from humans or other animal species than pigs. This caveat is illustrated by the fact that two previous studies did not find any significant differences between the performance of the two methods when analysing samples from poultry and cattle in Belgium [18,19]. Thus, there is an urgent international need to re-evaluate the performance of the 2-S method, as well as alternatives such as the 1-S method. This could, for example, be conducted within the EURL-AR network through proficiency testing.
